# Consumption environment, fan engagement, and social diffusion in non-professional football: an S-O-R study of China's Su Chao league

**DOI:** 10.3389/fspor.2026.1868151

**Published:** 2026-10-08

**Authors:** Mingxuan Xu, Wei He, Lai Wei

**Affiliations:** Department of Physical Education, Nanjing University, Nanjing, Jiangsu, China

**Keywords:** consumer satisfaction, emotional attachment, non-professional football league, social diffusion, S-O-R framework

## Abstract

**Introduction:**

Non-professional sports leagues represent an increasingly significant segment of the sports economy, yet the micro-level behavioral mechanisms through which innovative consumption environments generate sustained consumer engagement remain poorly understood. This study applies the Stimulus–Organism–Response (S-O-R) framework to the Jiangsu Provincial Super League (Su Chao), extending the stimulus dimension to capture the distinctive characteristics of culturally embedded grassroots sporting events, including civic identity, “one-city-one-team” rivalry, and industry integration.

**Methods:**

Survey data were collected from 382 Su Chao consumers. Confirmatory factor analysis and structural equation modeling were conducted together with bootstrap-based mediation and multi-group moderation tests. The stimulus tier was operationalized across four dimensions—scene immersion, industry integration, digital services, and basic facilities—while the organism tier was structured as a three-stage sequential system.

**Results:**

All 22 hypothesized structural paths were supported. Digital services and scene immersion emerged as the strongest stimulus predictors, whereas basic facilities functioned primarily as hygiene factors. Stimulus effects on behavioral responses were fully mediated through organism states. Participation frequency and consumer age significantly conditioned key pathways. The broad support for the hypothesized relationships should be interpreted in the context of Su Chao's highly anticipated inaugural season, characterized by novelty and high community engagement.

**Discussion:**

The findings indicate that digital services can supersede the physical environment as a primary driver of consumer experience in non-professional football. The study extends the S-O-R framework by demonstrating how culturally embedded consumption environments translate into sustained consumer engagement through sequential cognitive and affective mechanisms.

## 1 Introduction

### 1.1 Research background

The commercialization of spectator sport has long been studied through the lens of professional leagues and elite competition, yet non-professional and community-based sporting competitions are emerging as significant drivers of local consumer spending, urban identity formation, and social media traffic, challenging the assumption that economic value in sport flows primarily from the professional game (1, 2). Nowhere is this transformation more visible than in China, where city-level amateur football leagues erupted in 2025 to produce audience figures and consumption spillovers that rivaled many professional events. The Jiangsu Provincial Super League—popularly known as Su Chao (苏超)—exemplifies this phenomenon with unusual clarity. Launched in May 2025 under the joint auspices of the Jiangsu Provincial Sports Bureau and thirteen municipal governments, the league places one representative team in each of Jiangsu's prefecture-level cities, embedding civic identity, inter-city rivalry, and municipal pride constitutively in the competition format (3). Attendances regularly exceeded those of professional Chinese Super League fixtures held in the same cities during the same period, and the league generated over 2.3 billion social media impressions in its first season (4). These figures are all the more striking because Su Chao charges modest admission prices, offers no professional athletes, and allocates no broadcast rights revenue—a financial profile that sits entirely outside the conventional sports business model. The economic logic of Su Chao rests not on gate receipts or media rights but on a consumption ecosystem extending well beyond the stadium. By integrating match-day spectating with cultural tourism, intangible heritage experiences, local gastronomy, and digital fan engagement, the league has constructed what Chen et al. (5) term an innovative consumption scenario—a multi-dimensional service environment that converts attendance into a broader urban consumption encounter. This model has generated substantial spillovers into hospitality, retail, transportation, and creative industries in host cities (3). The operative mechanism, as theorized by Chai and Ye (6), is a traffic re-circulation dynamic: initial event traffic is converted through high-quality experiential engagement into durable consumer loyalty and peer-driven recruitment, generating a self-reinforcing cycle of attendance, advocacy, and repeat consumption. This dynamic unfolds against the backdrop of rising disposable incomes, a maturing middle class with strong preferences for experiential over material consumption, and the pervasive penetration of social media into leisure decision-making—conditions that collectively elevate emotional resonance, social identity expression, and shareable experience as primary drivers of consumption choices (5).

### 1.2 Research gaps

Despite the empirical salience of the Su Chao phenomenon, the academic literature has yet to provide an adequate theoretical account of the behavioral mechanisms that underlie it. Three interrelated gaps motivate the present study.

First, the behavioral mechanisms driving sustained consumer engagement in non-professional sports leagues remain theoretically underspecified. Existing research on non-professional sport has focused predominantly on participation motives, volunteer management, and social capital outcomes (1, 7), with limited attention to spectator consumption dynamics. Studies that have examined non-professional league spectatorship (2, 8) have identified the failure to sustain attendance beyond the initial novelty effect as a critical vulnerability but have not provided a systematic account of the psychological mechanisms through which initial positive experiences are converted into repeat attendance and peer advocacy. Second, the application of the Stimulus–Organism–Response (S-O-R) framework to sports event contexts has not kept pace with the complexity of contemporary consumption environments. Prior S-O-R applications in sport have predominantly operationalized the stimulus component in terms of services cape attributes—venue aesthetics, ambient conditions, and layout (9, 10)—neglecting the cultural programming, cross-industry service bundles, and digital ecosystems that define the distinctive value proposition of emerging non-professional leagues. Furthermore, the response component in extant sports S-O-R studies has been confined to conventional behavioral intentions that do not encompass the social diffusion behaviors central to the traffic economy logic through which Su Chao generates sustained growth. Third, the sport-tourism-cultural heritage fusion model that characterizes Su Chao lacks adequate behavioral explanation at the consumer level. Existing research on sport-tourism linkages has examined destination image, travel motivation, and aggregate spending patterns (2, 11) but has not systematically modeled the psychological pathway from specific fusion stimulus attributes to affective bonding and behavioral response at the individual consumer level. The cultural identity dimension of Su Chao—specifically, the activation of municipal pride and regional belonging through the one-city-one-team format—generates emotional responses that the services cape and experience quality constructs of conventional sports consumer behavior models are not designed to capture (12).

The cultural identity dimension of Su Chao—particularly the activation of municipal pride and regional belonging through the “one-city-one-team” format—elicits psychological responses that conventional sports consumer behavior models, which primarily emphasize servicescapes and experience quality, are not designed to capture. Because existing research has largely overlooked the roles of civic identity, cultural embeddedness, and cross-industry integration in shaping sports spectatorship, conventional frameworks struggle to fully explain consumer activation in non-professional sporting events. To address this gap, the present study explicitly incorporates these contextual characteristics into the stimulus dimension of the S-O-R framework. Specifically, Scene Immersion and Industry Integration are conceptualized as two context-specific stimulus dimensions that connect macro-level civic and cultural characteristics with consumers’ micro-level psychological responses, thereby extending the applicability of the S-O-R framework to grassroots sporting events.

### 1.3 Theoretical framework

#### 1.3.1 The S-O-R framework

The Stimulus–Organism–Response (S-O-R) framework, originally proposed by Mehrabian and Russell (13) within environmental psychology, offers a foundational theoretical architecture for understanding how external environmental stimuli shape consumer behavior through intervening psychological states. The framework posits that stimuli in the ambient environment trigger internal organism states—comprising cognitive evaluations and affective responses—which in turn drive behavioral reactions. This tripartite structure makes the S-O-R model particularly well suited to contexts where consumer behavior is mediated by experiential and emotional processes rather than by purely rational decision-making.

Since its introduction, the framework has been extensively applied across retail environments (14), online shopping (15), mobile commerce (16), social commerce (17), and experiential tourism (18). Within sport and leisure research, Pang et al. (19) demonstrated that service empathy perceptions generate memorable experiences that subsequently drive online word-of-mouth; Liu and Shi (20) found that perceived risk stimuli shaped motivation and attitude states that influenced behavioral intentions during the COVID-19 pandemic; and Jia et al. (21) revealed that experiential value perceptions mediate the path from cultural stimuli to shopping behavioral intentions. Two gaps persist across this body of work: the stimulus component has been operationalized in a unidimensional or at most bidimensional fashion that fails to capture the multi-layered nature of emerging non-professional sports leagues, and the response component has been largely confined to conventional behavioral intentions without incorporating social diffusion behaviors central to traffic economy logic. The present study addresses both gaps.

#### 1.3.2 Stimulus: the innovative consumption environment

Within the S-O-R framework, the stimulus component captures the external environmental attributes that initiate consumer psychological activation. Drawing on Chen et al.'s (5) conceptual work on innovative sports consumption scenarios, this study operationalizes the stimulus tier across four dimensions.

Scene immersion refers to the degree to which the event environment engulfs the consumer in a rich, multi-sensory experience through atmospheric richness, crowd choreography, interactive fan zones, and the integration of local cultural symbols and community narratives. Research consistently demonstrates that atmospheric richness and cultural embeddedness are primary antecedents of positive affective states at sports events (10, 22), and Su Chao exemplifies this dimension through its systematic embedding of intangible cultural heritage elements and municipal identity symbols across the event experience. Industry integration captures the breadth and coherence of the cross-sector consumption bundle offered beyond the core sporting spectacle—specifically, the linkage of sports viewing with cultural tourism, intangible heritage interactive experiences, and derivative merchandise. The convergence of sports with cultural tourism is recognized as a significant driver of destination attractiveness and consumer spending (11), while Zhang and Zhang (3) document how Su Chao's integration of sport, tourism, heritage, and commerce generates spatial spillover effects that extend value well beyond match day.

Digital services reflect the quality, convenience, and interactivity of the online ecosystem supporting and extending the consumption experience, encompassing ticketing platform efficiency, live streaming quality, online fan interaction features, and digital customer service responsiveness. The growing importance of digital service quality as a consumer experience driver is well documented in sports marketing research (23) and the broader e-service quality literature (24), and is especially salient for Su Chao's digitally native audience base (6). Basic facilities capture the foundational service infrastructure underpinning safety, accessibility, and price-fairness, including venue safety standards, staff professionalism, ticket and merchandise pricing policy, and physical accessibility. While less distinctive than the preceding dimensions, research consistently demonstrates that deficiencies in basic service provision suppress the positive effects of more experiential stimuli (25), making basic facility adequacy a necessary baseline condition for higher-order stimulus dimensions to operate effectively.

#### 1.3.3 Organism: cognitive and affective responses

The organism component represents the internal psychological states mediating the relationship between environmental stimuli and behavioral outcomes. Consistent with dual-process frameworks (26), the organism tier is structured as a three-stage cognitive-affective progression: experience perception, emotional attachment, and consumption satisfaction.

Experience perception refers to the consumer's rational cognitive appraisal of overall experience quality and value, encompassing evaluations of richness, service convenience, scene uniqueness, and value for money. Drawing on service quality research (27) and experience economy theory (28), cognitive quality assessments are positioned as primary antecedents of behavioral outcomes. Yoshida and James (22) demonstrate that perceived service quality and game quality evaluations jointly predict fan satisfaction, while Jia et al. (21) confirm that utilitarian and hedonic experiential value perceptions mediate the path from cultural heritage stimuli to consumer behavioral intentions.

Emotional attachment captures the affective bonding between the consumer and the event, its associated cultural identity, and its brand. Drawing on place attachment theory (29) and brand affect research (30), this construct encompasses urban identity reinforcement, positive emotional identification with the event, subjective enjoyment, and favorable brand attitude. Sui et al. (12) demonstrate that Su Chao's construction of urban honor rituals activates geographically grounded identity mechanisms generating strong emotional bonding between consumers and their home-city teams, creating affective states qualitatively distinct from those generated by conventional sports consumption. Emotional attachment to sports entities functions as a powerful driver of loyalty behaviors relatively resistant to performance fluctuations (31).

Consumption satisfaction represents the consumer's integrative evaluative judgment, synthesizing cognitive quality appraisals and affective experiences into an overall assessment of whether the consumption encounter fulfilled expectations. Following Oliver's (32) expectancy-disconfirmation model, satisfaction is conceptualized as a post-experience evaluation reflecting the perceived gap between expected and delivered value, and has been consistently identified as the most proximal determinant of repeat attendance and behavioral loyalty in sports consumption research (33). The positioning of satisfaction as the final organism stage—downstream of both experience perception and emotional attachment—enables the study to test the serial mediation chain through which stimuli ultimately reach behavioral outputs.

#### 1.3.4 Response: re-consumption intention and traffic circulation behavior

The response component captures the behavioral outputs triggered by organism states. Beyond conventional approach behaviors, this study introduces Traffic Circulation Behavior to capture the social diffusion functions central to the traffic economy logic. It is crucial to distinguish this construct from traditional word-of-mouth (WOM). While traditional WOM often relies on passive, offline interpersonal recommendations, traffic circulation behavior encompasses active digital traffic diffusion, highly visible social media sharing, and structured peer mobilization within online and offline communities (6). This active recirculation converts highly satisfied consumers into traffic-generating nodes.

### 1.4 Hypotheses development

#### 1.4.1 Stimulus effects on organism states (H1–H4)

Rather than detailing identical pathways for each stimulus, we conceptualize that the four stimulus dimensions collectively but distinctly elevate consumer psychological states. Scene immersion and industry integration primarily enhance the experiential richness and cultural embeddedness of the event, thereby heavily influencing cognitive quality appraisals and affective identity bonding (12). Digital services act as a critical touchpoint that reduces friction and fulfills the interactivity expectations of modern consumers, directly driving cognitive and emotional evaluations. Basic facilities function as foundational hygiene factors; while they may not drive profound emotional attachment, they are prerequisites for overall satisfaction (25).

H1–H4: Scene immersion (H1), industry integration (H2), digital services (H3), and basic facilities (H4) each positively influence experience perception, emotional attachment, and consumption satisfaction.

#### 1.4.2 Organism effects on behavioral responses (H5–H11)

Consistent with dual-process frameworks (26), cognitive evaluation precedes affective bonding. Thus, we hypothesize that experience perception drives both emotional attachment and integrative consumption satisfaction (H5). Furthermore, these internal states are theorized to dictate behavioral responses. Experience perception and emotional attachment provide the rational and identity-driven foundations for loyalty, while consumption satisfaction serves as the most proximal catalyst for both re-consumption and traffic circulation behaviors (H6–H8). Consequently, the organism tier acts as a mediating mechanism. The S-O-R architecture dictates that external stimuli do not drive behavior directly but are fully mediated by internal cognitive and affective states (H9–H10), culminating in a sequential serial mediation chain from cognition to affect to satisfaction (H11).

H5: Experience perception positively influences emotional attachment (H5a) and consumption satisfaction (H5b).
H6: Experience perception positively influences re-consumption intention (H6a) and traffic circulation behavior (H6b).
H7: Emotional attachment positively influences re-consumption intention (H7a) and traffic circulation behavior (H7b).
H8: Consumption satisfaction positively influences re-consumption intention (H8a) and traffic circulation behavior (H8b).
H9: Experience perception mediates the positive relationship between each stimulus dimension and each behavioral response.
H10: Emotional attachment mediates the positive relationship between each stimulus dimension and each behavioral response.
H11: Consumption satisfaction serially mediates the relationship between experience perception, emotional attachment, and behavioral response, such that stimulus effects are transmitted through the sequential chain: experience perception→emotional attachment→consumption satisfaction→response.

#### 1.4.3 Moderation effects of participation frequency and age (H12–H13)

Drawing on habit formation and identity consolidation literature, we posit that event participation frequency moderates the relationship between emotional attachment and re-consumption. As consumers repeatedly engage with the event, their psychological connection to the “one-city-one-team” civic identity consolidates, transforming transient emotional arousal into durable behavioral loyalty (31).

Furthermore, digital sports consumption literature highlights significant generational heterogeneity in technology adoption and e-service reliance. Younger “digital native” consumers are highly sensitive to digital service interfaces, relying on ticketing efficiency and online interactivity as primary lenses for evaluating overall event quality, whereas older cohorts may prioritize physical touchpoints (23).

H12: Event participation frequency positively moderates the relationship between emotional attachment (O2) and re-consumption intention (R1).
H13: Respondent age moderates the relationship between digital services (S3) and experience perception (O1), such that the effect is stronger for younger consumers.

## 2 Methodology

### 2.1 Conceptual model

Grounded in the Stimulus–Organism–Response (S-O-R) framework (13), this study proposes an integrated conceptual model to explain how the distinctive consumption environment of the Su Chao League shapes consumers’ psychological responses and ultimately drives behavioral outcomes. The model is organized into three hierarchical tiers corresponding to the core components of the S-O-R framework.

The Stimulus (S) tier comprises four context-specific dimensions that capture the multi-layered nature of the Su Chao consumption environment: (1) Scene Immersion, reflecting the atmospheric, cultural, and identity-related characteristics of the event; (2) Industry Integration, capturing the integration of sports, tourism, and local cultural resources; (3) Digital Services, representing the accessibility and interactivity of digital platforms; and (4) Basic Facilities, encompassing physical infrastructure, safety, and pricing fairness. By incorporating Scene Immersion and Industry Integration as context-specific stimulus dimensions, the model extends the applicability of the S-O-R framework to grassroots sporting events characterized by civic identity and cultural embeddedness. The Organism (O) tier includes three interrelated psychological states: Experience Perception, Emotional Attachment, and Consumption Satisfaction, representing consumers’ cognitive, affective, and evaluative responses to the event. The Response (R) tier consists of two complementary behavioral outcomes: Re-consumption Intention and Traffic Circulation Behavior. The latter is defined as consumers’ active dissemination of event-related content through social media sharing, word-of-mouth recommendation, and peer mobilization, through which consumers become active participants in the circulation of attention and local economic value.

Together, the proposed model specifies the direct effects of stimulus dimensions on psychological responses, the subsequent influences of organism variables on behavioral outcomes, as well as mediation and moderation mechanisms that explain how the Su Chao consumption environment translates into sustained consumer engagement. The conceptual framework is illustrated in Figure 1, and the complete set of hypotheses is presented in Table 1.

**Figure 1:**
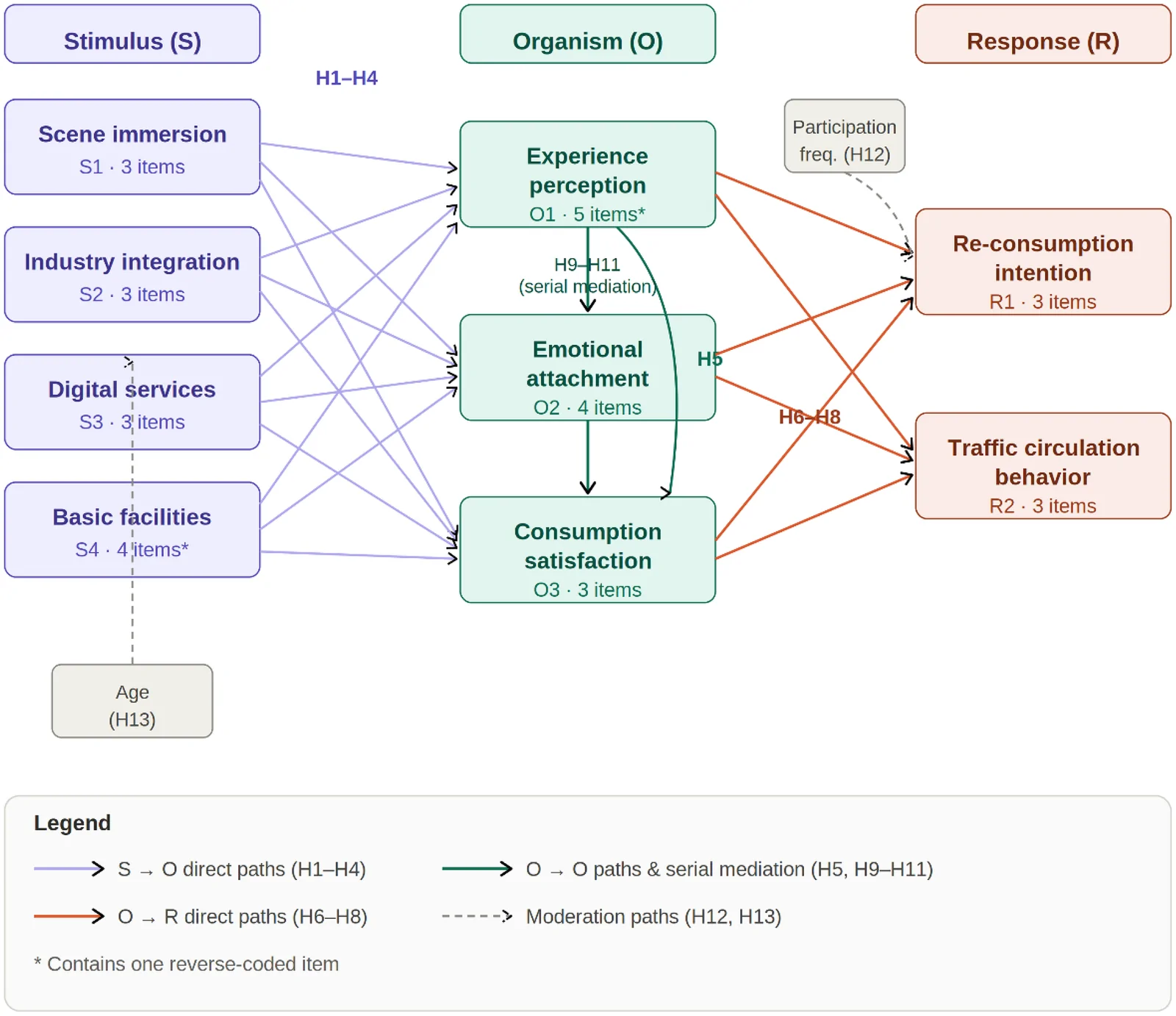
Conceptual model based on the S-O-R framework.

**Table 1:** Summary of research hypotheses.

Hypothesis	Statement	Path Type
H1	Scene immersion (S1) positively influences experience perception (O1), emotional attachment (O2), and consumption satisfaction (O3).	S→O
H2	Industry integration (S2) positively influences experience perception (O1), emotional attachment (O2), and consumption satisfaction (O3).	S→O
H3	Digital services (S3) positively influences experience perception (O1), emotional attachment (O2), and consumption satisfaction (O3).	S→O
H4	Basic facilities (S4) positively influences experience perception (O1), emotional attachment (O2), and consumption satisfaction (O3).	S→O
H5	Experience perception (O1) positively influences emotional attachment (O2) and consumption satisfaction (O3).	O→O
H6	Experience perception (O1) positively influences re-consumption intention (R1) and traffic circulation behavior (R2).	O→R
H7	Emotional attachment (O2) positively influences re-consumption intention (R1) and traffic circulation behavior (R2).	O→R
H8	Consumption satisfaction (O3) positively influences re-consumption intention (R1) and traffic circulation behavior (R2).	O→R
H9	Experience perception (O1) mediates the relationship between stimulus dimensions and behavioral responses.	Mediation
H10	Emotional attachment (O2) mediates the relationship between stimulus dimensions and behavioral responses.	Mediation
H11	Consumption satisfaction (O3) acts as a serial mediator between experience perception/emotional attachment and behavioral responses.	Serial Mediation
H12	Event participation frequency moderates the relationship between emotional attachment (O2) and re-consumption intention (R1).	Moderation
H13	Respondent age moderates the relationship between digital services (S3) and experience perception (O1).	Moderation

All hypothesized relationships are directional (positive). S, Stimulus; O, Organism; R, Response.

### 2.2 Measurement development

All measurement items were developed through a two-stage process. First, a systematic review of validated scales from prior S-O-R applications in sports consumption and cultural tourism was conducted. Stimulus dimensions drew on the services cape literature (14) and recent studies of innovative sports consumption environments in China (3, 5, 6). Organism constructs were adapted from scales validated in event experience (19, 21), emotional attachment (34), and consumer satisfaction (32) research. Response items were drawn from word-of-mouth and repurchase intention scales (35, 36).

Second, all borrowed items were re-worded to reflect the specific sport-tourism-intangible heritage-digital service fusion context of Su Chao, and reviewed for content validity by two independent sports marketing researchers. This process yielded a 31-item instrument across nine constructs, measured on a 5-point Likert scale (1 = strongly disagree, 5 = strongly agree). Two reverse-coded items were included to reduce acquiescence bias (S4_4 and O1_5). A pilot study (*N* = 50) was conducted with participants from Su Chao online communities to confirm the instrument's psychometric properties. The results demonstrated satisfactory reliability and validity: all items exhibited item-total correlations >.50, exploratory factor loadings ranged from .68 to .85, and construct Cronbach's *α* values ranged from .78 to .88, confirming satisfactory internal consistency. One Basic Facilities item was slightly reworded for clarity based on respondent feedback before full-scale data collection.

### 2.3 Data collection and sample

The target population comprised consumers who had engaged with the Su Chao league either through on-site attendance or online viewership. This dual framing was adopted deliberately: because Su Chao's consumption model explicitly integrates physical spectating with digital participation (live streaming, social media interaction, virtual merchandise), restricting the sample to on-site attendees would systematically exclude a substantial segment of the event's traffic-generating audience. Data were collected through a mixed-mode survey design combining online and offline channels. Online distribution was conducted via the official Su Chao social media communities, ticketing platform user databases, and targeted posts on Weibo and WeChat during the 2025 Su Chao season (May–August 2025). Offline collection was carried out at match venues across Jiangsu Province, with trained research assistants administering paper questionnaires at entry gates, spectator rest areas, and merchandise stands. This multi-channel approach was adopted to mitigate single-source sampling bias and to ensure adequate representation of both digital-first and on-site consumer segments.

A total of 500 questionnaires were distributed. Data quality screening excluded respondents who (1) reported no prior awareness of the Su Chao league, (2) completed the questionnaire in less than three minutes (indicating careless responding), or (3) produced logically inconsistent response patterns (e.g., endorsing both a positively and negatively worded item at the same extreme level). Following this filtering procedure, 382 valid responses were retained, yielding an effective response rate of 76.4%. Based on the rule-of-thumb that structural equation modeling requires a minimum of 10 observations per estimated parameter (37), and given that the present model contains 31 observed variables, the achieved sample size is well in excess of the recommended minimum of 310 cases. Sample characteristics are reported in full in the Results section (Table 1). Briefly, the sample is predominantly young and well-educated, with 41.38% of respondents aged 19–25 and 89.65% holding a bachelor's degree or higher. Monthly income is concentrated in the 5,001–12,000 RMB range (62.07%), reflecting a middle-income consumer profile consistent with the broader Su Chao audience demographic.

### 2.4 Variables and measures

Table 2 provides an overview of all constructs, their dimensionality, item counts, and representative items. A detailed description of each construct follows. Stimulus (S). The stimulus tier captures external environmental and service provision factors that serve as the initiating conditions for consumer psychological activation. Four sub-dimensions are specified. Scene Immersion (S1, 3 items) assesses the degree to which Su Chao creates an atmospherically rich and culturally embedded viewing environment, including the on-site spectacle, local cultural symbol integration, and fan interaction design. Industry Integration (S2, 3 items) measures the depth of the sports-tourism-intangible heritage bundle, including the sport-tourism linkage, non-material cultural heritage interactive experiences, and derivative product attractiveness. Digital Services (S3, 3 items) captures the convenience, interactivity, and quality of Su Chao's online ecosystem, including ticketing efficiency, interactive function completeness, and overall digital experience enhancement. Basic Facilities (S4, 4 items, including one reverse-coded) assesses the foundational service environment: safety, staff professionalism, price fairness, and physical accessibility.

**Table 2:** Construct definitions, dimensionality, and measurement items.

Construct	Dimension	Items (*N*)	Sample Item	Source
Stimulus (S)	Scene Immersion (S1)	3	"Su Chao creates an immersive viewing atmosphere with strong local cultural elements."	Adapted from Chen et al. (2025) (5)
	Industry Integration (S2)	3	"Su Chao provides a comprehensive experience combining sports viewing and cultural tourism."	Adapted from Zhang & Zhang (2025) (3)
	Digital Services (S3)	3	"The online ticketing and viewing processes of Su Chao are convenient and efficient."	Adapted from Chai & Ye (2026) (6)
	Basic Facilities (S4)	4*	"Su Chao provides a safe and reliable spectating and consumption environment."	Adapted from Donovan & Rossiter (1982) (14)
Organism (O)	Experience Perception (O1)	5*	"The overall Su Chao experience is rich and appealing."	Adapted from Jia et al. (2022) (21)
	Emotional Attachment (O2)	4	"Participating in Su Chao has strengthened my sense of identity with the city."	Adapted from Cheng & Yuan (2026) (34)
	Consumption Satisfaction (O3)	3	"The overall Su Chao experience met my expectations."	Adapted from Pang et al. (2025) (19)
Response (R)	Re-consumption Intention (R1)	3	"I am willing to participate in Su Chao-related activities again."	Adapted from Cheng & Huang (2024) (35)
	Traffic Circulation Behavior (R2)	3	"I am willing to recommend Su Chao experiences to others."	Adapted from Li & Guo (2025) (36)

* indicates that the dimension contains one reverse-coded item. All items were measured on a five-point Likert scale (1 = strongly disagree, 5 = strongly agree). Sources reflect the primary scale origins from which items were adapted for the SuChao context.

#### 2.4.1 Organism (O)

The organism tier is operationalized as a three-stage cognitive-affective progression. Experience Perception (O1, 5 items, including one reverse-coded) reflects the consumer's rational cognitive evaluation of the overall experience along four dimensions: richness, service convenience, scene uniqueness, and value for money. Emotional Attachment (O2, 4 items) captures affective bonding along four facets: urban identity reinforcement, positive emotional identification with the event, subjective enjoyment and engagement, and favorable brand attitude toward Su Chao. Consumption Satisfaction (O3, 3 items) measures the consumer's integrative evaluative judgment of the overall experience, assessing expectation fulfillment, service quality satisfaction, and global satisfaction.

#### 2.4.2 Response (R)

The response tier comprises two behavioral constructs. Re-consumption Intention (R1, 3 items) measures the consumer's willingness to re-attend, continue following, and repurchase Su Chao-related products and services. Traffic Circulation Behavior (R2, 3 items) operationalizes the word-of-mouth and social diffusion functions that are central to the traffic re-circulation mechanism: willingness to recommend to others, willingness to share experiences on social media, and willingness to mobilize peer participation.

### 2.5 Data analysis strategy

Data analysis proceeded in five sequential stages, implemented in SPSS 26.0 and Amos 24.0. First, descriptive statistics were computed for all study variables and demographic characteristics. Means, standard deviations, and bivariate correlations among the six main constructs were examined to assess data distributions and preliminary inter-construct associations.

Second, the measurement model was assessed through a two-step procedure (38). An exploratory factor analysis (EFA) using principal component extraction with Varimax rotation was first conducted to verify the factor structure of the stimulus tier, given the four-dimensional specification of S that departs from the unidimensional treatment in prior studies. Items with factor loadings below 0.50 or cross-loadings above 0.35 were flagged for removal. Confirmatory factor analysis (CFA) was then performed using Amos 24.0 to evaluate the overall measurement model fit and to establish construct reliability and validity. Model fit was evaluated against conventional benchmarks: $\chi^{2}/df\;<\;3$, Comparative Fit Index (CFI) > 0.90, Tucker–Lewis Index (TLI) > 0.90, Root Mean Square Error of Approximation (RMSEA) < 0.08, and Standardized Root Mean Square Residual (SRMR) < 0.08 (39). Internal consistency reliability was assessed via Cronbach's *α* (threshold ≥ 0.70) and composite reliability (CR, threshold ≥0.70). Convergent validity was evaluated using average variance extracted [AVE, threshold ≥0.50; (40)]. Discriminant validity was confirmed by verifying that the square root of each construct's AVE exceeded its highest inter-construct correlation.

Third, the structural model was estimated to test the direct hypotheses (H1–H8). Standardized path coefficients (*β*), critical ratios (C.R.), and associated *p*-values were reported for all hypothesized paths. The overall structural model fit was evaluated using the same indices as the measurement model. Fourth, mediation hypotheses (H9–H11) were tested using bias-corrected bootstrap confidence intervals (5,000 resamples) as implemented in Amos 24.0 (41). For each proposed mediation path, indirect effects were considered significant if the 95% bias-corrected bootstrap confidence interval excluded zero. Serial mediation through the O1→O2→O3 chain was tested separately, and the proportion of total effect mediated was calculated to evaluate the relative importance of each indirect pathway.

Fifth, moderation hypotheses (H12 and H13) were tested using multi-group structural equation modeling (MG-SEM). For H12, the sample was split into first-time participants (those reporting one or zero prior Su Chao participations) and repeat participants (two or more participations). For H13, respondents were divided into a younger group (aged 18–35) and an older group (aged 36 and above). The configural model (no equality constraints) was first estimated to verify acceptable fit in both subgroups. A series of nested models with progressively tighter equality constraints (metric invariance, scalar invariance) was then compared using the *χ*^2^ difference test (*Δχ*^2^, *p* < 0.05) and the CFI change criterion [*Δ*CFI < 0.010; (42)]. A significant difference in the focal path coefficient between subgroups constituted support for the moderation hypothesis.

To address the potential threat of common method bias (CMB) arising from the single-source, self-report survey design, Harman's single-factor test was performed. An unrotated principal component analysis of all scale items was conducted; if a single factor accounted for the majority of the total variance, CMB would be considered a serious concern (43). In addition, the marker variable technique was applied as a supplementary check, with one theoretically unrelated item included in the survey to serve as a proxy for common method variance.

## 3 Results

### 3.1 Descriptive statistics

A total of 500 questionnaires were distributed; following exclusion of respondents with no prior league awareness, implausibly short completion times, or logically inconsistent response patterns, 382 valid responses were retained (effective response rate = 76.4%). Based on the rule-of-thumb of 10 observations per estimated parameter (37), this sample size well exceeds the recommended minimum of 310 cases for the present model.

The sample is predominantly male (56.81%) and young, with 41.38% aged 19–25. Educational attainment is high, with 89.65% holding a bachelor's degree or above. Monthly income is concentrated in the 5,001–12,000 RMB range (62.07%), reflecting a middle-income consumer profile consistent with the broader Su Chao audience demographic. Full sample characteristics are reported in Table 3. From a behavioral perspective, engagement is moderate: 39.88% of respondents participated in football-related activities 1–2 times in the past year, and only 7.25% reported high-frequency participation (≥11 times). Familiarity with non-professional leagues was similarly distributed, with 41.21% reporting a neutral level—indicating that the sample captures a broad spectrum of consumer awareness rather than a niche enthusiast group.

**Table 3:** Sample characteristics (*N* = 382).

Panel A: Demographic Characteristics			
Variable	Category	Frequency	Percentage (%)
Gender	Male	217	56.81
	Female	165	43.19
Age	≤18	13	3.45
	19–25	158	41.38
	26–35	79	20.69
	36–45	79	20.69
	≥46	53	13.79
Education	High school or below	40	10.34
	Bachelor	211	55.17
	Master+	131	34.48
Monthly income	≤3000	26	6.9
	3001–5000	53	13.79
	5001–8000	105	27.59
	8001–12000	132	34.48
	≥12001	66	17.24

Panel B: Participation and Behavioral Characteristics			
Variable	Category	Frequency	Percentage (%)
Participation Frequency in Football Activities (Past Year)	Never	51	13.4
	1–2 times	152	39.88
	3–5 times	92	24.17
	6–10 times	58	15.3
	≥11 times	28	7.25
Familiarity with Non-professional Football Leagues	Very unfamiliar	40	10.49
	Not very familiar	93	24.39
	Neutral	157	41.21
	Familiar	62	16.34
	Very familiar	29	7.57

Table 4 presents descriptive statistics and the inter-construct correlation matrix. Satisfaction (O3) recorded the highest mean (M = 4.10), followed by Stimulus (M = 3.90), Experience Perception and behavioral responses (M = 3.80), and Emotional Attachment (M = 3.70). The relatively lower emotional attachment score suggests room to further deepen affective consumer connections despite generally positive functional evaluations. All inter-construct correlations are positive and moderate, supporting the theoretical relationships proposed in the S-O-R framework without raising multicollinearity concerns. Discriminant validity is confirmed, with the square root of each construct's AVE exceeding its highest inter-construct correlation.

**Table 4:** Descriptive statistics, reliability, and discriminant validity (*N* = 382).

Construct	Mean	SD	S	O1	O2	O3	R1	R2
S(Stimulus)	3.90	0.76	**0** **.** **78**					
O1(Experience)	3.80	0.81	0.63	**0**.**76**				
O2(Emotion)	3.70	0.92	0.52	0.51	**0**.**79**			
O3(Satisfaction)	4.10	0.73	0.55	0.57	0.55	**0**.**80**		
R1(Re-consumption)	3.80	0.81	0.48	0.50	0.52	0.63	**0**.**81**	
R2(Social diffusion)	3.80	0.80	0.45	0.48	0.50	0.52	0.58	**0.77**

Diagonal elements (in bold) represent the square root of the Average Variance Extracted ($\sqrt{AVE}$). Off-diagonal elements represent Pearson correlation coefficients.

### 3.2 Measurement model assessment

The measurement model was assessed using confirmatory factor analysis (CFA) conducted in Amos 24.0 to evaluate the reliability and validity of all nine constructs. The results are presented in Table 5 (1). Internal consistency reliability. The Cronbach's *α* values for all constructs ranged from 0.85 to 0.90, well above the recommended threshold of 0.70 (44). Composite reliability (CR) values ranged from 0.88 to 0.92, further confirming satisfactory construct reliability. Both indices indicate that the items within each construct are measuring a coherent underlying factor (2). Convergent validity. All standardized factor loadings ranged from 0.76 to 0.88 and were statistically significant (*p* < 0.001), indicating that the observed variables adequately represent their respective latent constructs (37). The AVE values for all constructs ranged from 0.65 to 0.79, all exceeding the recommended threshold of 0.50 (40), confirming that each construct accounts for a majority of the variance in its indicators. Taken together, these results provide strong evidence of convergent validity across all nine constructs (3). Discriminant validity. Discriminant validity was assessed using the Fornell–Larcker criterion (40). As reported in Table 5, the square root of the AVE for each construct exceeds the highest inter-construct correlation in the corresponding row and column of the correlation matrix. This pattern of results demonstrates that each construct shares more variance with its own indicators than with any other construct in the model, confirming satisfactory discriminant validity (4). Common method bias. Given the single-source, self-report nature of the survey data, Harman's single-factor test was conducted to assess the potential influence of common method bias (43). An unrotated principal component analysis of all scale items yielded a first factor that accounted for 28.4% of the total variance—well below the 50% threshold that would indicate a serious common method bias problem. This result suggests that common method variance is unlikely to be a major confound in the present data. The measurement model demonstrates satisfactory reliability, convergent validity, and discriminant validity across all constructs, providing a solid psychometric foundation for the subsequent structural model analysis.

**Table 5:** Confirmatory factor analysis results: reliability and validity (*N* = 382).

Construct & Dimension	Item	Std. Loading (*λ*)	*α*	CR	AVE
**Stimulus (S)** *Scene Immersion (S1)*	S1_1	0.84	0.88	0.90	0.75
	S1_2	0.86			
	S1_3	0.82			
*Industry Integration (S2)*	S2_1	0.81	0.86	0.89	0.73
	S2_2	0.85			
	S2_3	0.83			
*Digital Services (S3)*	S3_1	0.80	0.87	0.90	0.75
	S3_2	0.87			
	S3_3	0.84			
*Basic Facilities (S4)*	S4_1	0.78	0.85	0.88	0.65
	S4_2	0.82			
	S4_3	0.80			
	S4_4r	0.76			
**Organism (O)** *Experience Perception (O1)*	O1_1	0.83	0.89	0.91	0.68
	O1_2	0.85			
	O1_3	0.81			
	O1_4	0.84			
	O1_5r	0.77			
*Emotional Attachment (O2)*	O2_1	0.86	0.90	0.92	0.73
	O2_2	0.88			
	O2_3	0.84			
	O2_4	0.82			
*Consumption Satisfaction (O3)*	O3_1	0.87	0.89	0.91	0.77
	O3_2	0.85			
	O3_3	0.88			
**Response (R)** *Re-consumption Intention (R1)*	R1_1	0.86	0.90	0.92	0.79
	R1_2	0.88			
	R1_3	0.84			
*Traffic Circulation (R2)*	R2_1	0.82	0.87	0.89	0.72
	R2_2	0.85			
	R2_3	0.83			

All standardized factor loadings are significant at *p* < .001. α = Cronbach's alpha; CR, composite reliability; AVE, average variance extracted. R, reverse-coded item (recoded prior to analysis as 6−original score). The CFA model demonstrated a good fit to the data, with $\;\chi^{2}/df=2.47$, CFI = 0.95, TLI = 0.94, RMSEA = 0.049, and SRMR = 0.041, indicating a good model fit according to commonly accepted thresholds in structural equation modeling.

### 3.3 Structural model results

The structural model was estimated using Amos 24.0 to test the hypothesized direct paths among the stimulus, organism, and response constructs. The model demonstrated satisfactory fit to the empirical data: $\chi^{2}/df=2.63$, CFI = 0.95, TLI = 0.94, RMSEA = 0.051, SRMR = 0.043. All indices meet or exceed the conventional thresholds recommended by Hu and Bentler (39), and the minor deterioration relative to the measurement model is expected when the full nomological network of structural paths is estimated. The standardized path coefficients and significance tests for all hypothesized relationships are reported in Table 6.

**Table 6:** Structural model path coefficients and hypothesis test results (*N* = 382).

Hyp.	Path	*β*	p	Supported?
Panel A: Stimulus→Organism (H1–H4)				
H1a	Scene Immersion (S1)→Experience Perception (O1)	0.34	<0.001	✓ Supported
H1b	Scene Immersion (S1)→Emotional Attachment (O2)	0.31	<0.001	✓ Supported
H1c	Scene Immersion (S1)→Consumption Satisfaction (O3)	0.29	<0.001	✓ Supported
H2a	Industry Integration (S2)→Experience Perception (O1)	0.27	<0.001	✓ Supported
H2b	Industry Integration (S2)→Emotional Attachment (O2)	0.25	<0.01	✓ Supported
H2c	Industry Integration (S2)→Consumption Satisfaction (O3)	0.23	<0.01	✓ Supported
H3a	Digital Services (S3)→Experience Perception (O1)	0.36	<0.001	✓ Supported
H3b	Digital Services (S3)→Emotional Attachment (O2)	0.33	<0.001	✓ Supported
H3c	Digital Services (S3)→Consumption Satisfaction (O3)	0.35	<0.001	✓ Supported
H4a	Basic Facilities (S4)→Experience Perception (O1)	0.19	<0.01	✓ Supported
H4b	Basic Facilities (S4)→Emotional Attachment (O2)	0.21	<0.01	✓ Supported
H4c	Basic Facilities (S4)→Consumption Satisfaction (O3)	0.18	<0.05	✓ Supported
Panel B: Organism→Organism (H5)				
H5a	Experience Perception (O1)→Emotional Attachment (O2)	0.42	<0.001	✓ Supported
H5b	Experience Perception (O1)→Consumption Satisfaction (O3)	0.28	<0.001	✓ Supported
—	Emotional Attachment (O2)→Consumption Satisfaction (O3)	0.39	<0.001	✓ Supported
Panel C: Organism→Response (H6–H8)				
H6a	Experience Perception (O1)→Re-consumption Intention (R1)	0.31	<0.001	✓ Supported
H6b	Experience Perception (O1)→Traffic Circulation Behavior (R2)	0.26	<0.001	✓ Supported
H7a	Emotional Attachment (O2)→Re-consumption Intention (R1)	0.37	<0.001	✓ Supported
H7b	Emotional Attachment (O2)→Traffic Circulation Behavior (R2)	0.29	<0.001	✓ Supported
H8a	Consumption Satisfaction (O3)→Re-consumption Intention (R1)	0.45	<0.001	✓ Supported
H8b	Consumption Satisfaction (O3)→Traffic Circulation Behavior (R2)	0.34	<0.001	✓ Supported

Standardized path coefficients (β) are reported. All paths estimated simultaneously in a single structural model. Model fit: *χ*^2^/df = 2.63, CFI = 0.95, TLI = 0.94, RMSEA = 0.051, SRMR = 0.043. ✓ = hypothesis supported; *p*-values based on two-tailed tests.

#### 3.3.1 Stimulus effects on organism states (H1–H4)

All twelve hypothesized paths from the four stimulus dimensions to the three organism constructs were statistically significant and positive in direction, providing full support for H1 through H4. Among the stimulus dimensions, Digital Services (S3) emerged as the strongest predictor of Experience Perception (*β* = 0.36, *p* < .001) and Emotional Attachment (*β* = 0.33, *p* < .001), and the second strongest predictor of Consumption Satisfaction (*β* = 0.35, *p* < .001). Scene Immersion (S1) produced the largest effect on Consumption Satisfaction (*β* = 0.29, *p* < .001) and exerted substantial effects on both Experience Perception (*β* = 0.34, *p* < .001) and Emotional Attachment (*β* = 0.31, *p* < .001). Industry Integration (S2) demonstrated consistent moderate effects across all three organism constructs (*β* = 0.23–0.27), while Basic Facilities (S4) produced the weakest but still significant effects (*β* = 0.18–0.21), consistent with its theoretical role as a hygiene-level baseline condition rather than a primary driver of affective engagement.

#### 3.3.2 Organism-to-organism paths (H5)

Experience Perception (O1) exerted a strong positive effect on Emotional Attachment (O2) (*β* = 0.42, *p* < .001), supporting H5a and confirming that cognitive quality appraisals precede and inform affective bonding as theorized in the dual-process architecture of the organism tier. Experience Perception also produced a significant direct effect on Consumption Satisfaction (O3) (*β* = 0.28, *p* < .001), supporting H5b. Additionally, Emotional Attachment exerted a strong effect on Consumption Satisfaction (*β* = 0.39, *p* < .001), completing the sequential cognitive-affective-evaluative chain at the heart of the organism tier's theoretical structure.

#### 3.3.3 Organism effects on behavioral responses (H6–H8)

All six hypothesized paths from organism constructs to behavioral response variables were statistically significant and positive. Consumption Satisfaction (O3) was the strongest predictor of both Re-consumption Intention (*β* = 0.45, *p* < .001) and Traffic Circulation Behavior (*β* = 0.34, *p* < .001), supporting H8a and H8b and confirming satisfaction as the most proximal behavioral driver in the S-O-R chain. Emotional Attachment (O2) produced the second-largest effects on Re-consumption Intention (*β* = 0.37, *p* < .001) and Traffic Circulation Behavior (*β* = 0.29, *p* < .001), supporting H7a and H7b. Experience Perception (O1) also significantly predicted both response variables (R1: *β* = 0.31, *p* < .001; R2: *β* = 0.26, *p* < .001), supporting H6a and H6b. Notably, the effect of Emotional Attachment on Re-consumption Intention (*β* = 0.37) exceeded that of Experience Perception (*β* = 0.31), suggesting that affective bonding with the Su Chao event and its associated urban identity operates as a particularly potent motivator of repeat engagement.

All 22 hypothesized direct paths were supported at conventional significance thresholds (*p* < .05 or better), with the majority reaching *p* < .001. The pattern of results is consistent with the theoretical expectation that Consumption Satisfaction functions as the dominant proximal driver of behavioral response, while Digital Services and Scene Immersion are the most influential distal stimulus predictors. A complete summary of path coefficients and hypothesis test results is provided in Table 6, and the structural model with standardized path coefficients is illustrated in Figure 2.

**Figure 2:**
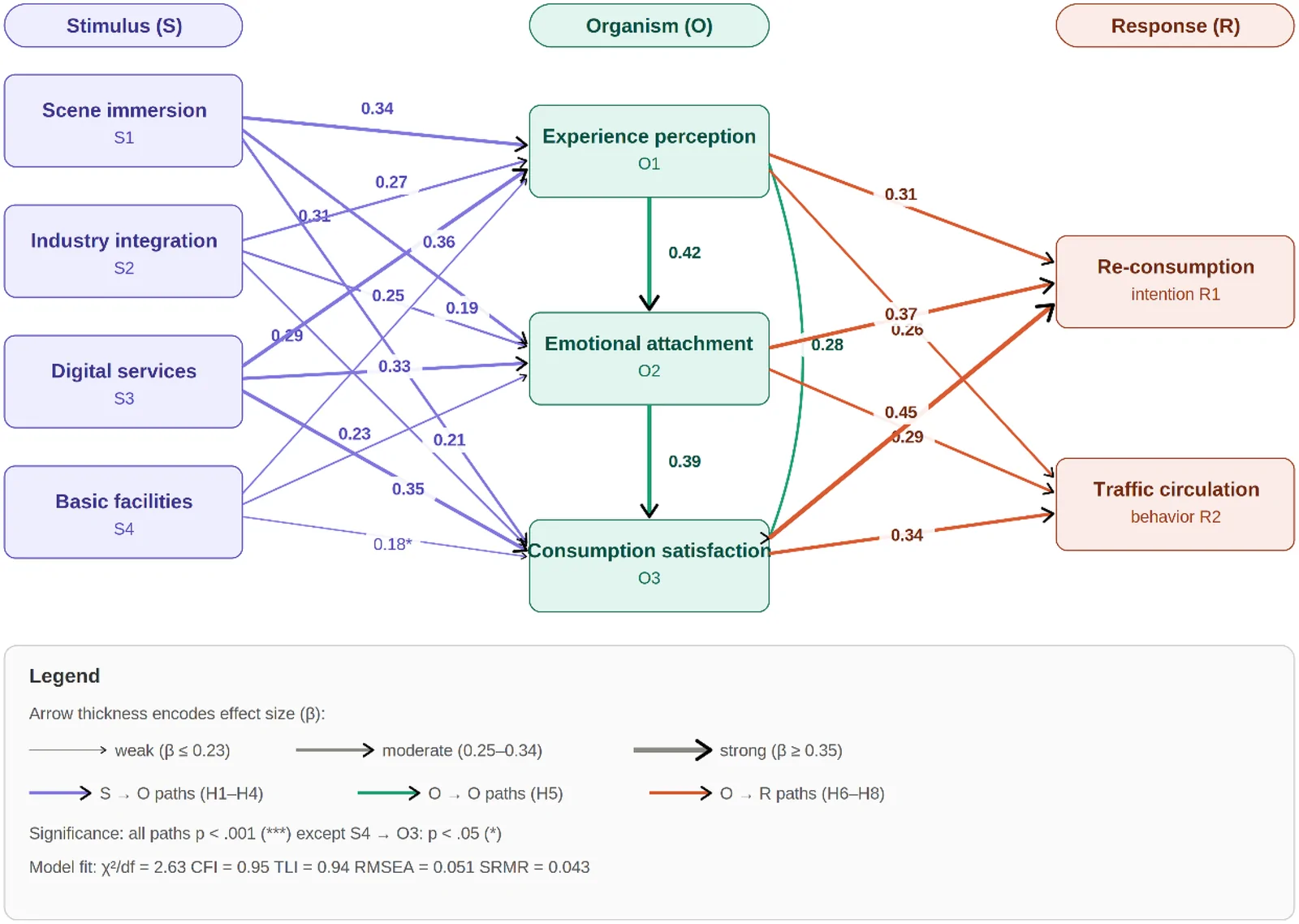
**Structural model results with standardized path coefficients (*N*** **=** **382).** Arrow thickness is proportional to the magnitude of the standardized path coefficient (*β*). All paths are statistically significant at *p* < 0.05 or better. The only path significant at the.05 level was S4→O3 (*β* = 0.18, *p* < 0.05). Model fit: *χ*^2^/df = 2.63, CFI = 0.95, TLI = 0.94, RMSEA = 0.051, SRMR = 0.043.

### 3.4 Mediation analysis

To test the mediation hypotheses (H9–H11), bias-corrected bootstrap confidence intervals based on 5,000 resamples were computed using the phantom variable approach implemented in Amos 24.0 (41). An indirect effect was considered statistically significant if the 95% bias-corrected confidence interval excluded zero. The full set of mediation results is presented in Table 7. The standardized indirect effects and their 95% bias-corrected bootstrap confidence intervals are visualized in Figure 3.

**Table 7:** Bootstrap mediation results (5,000 resamples, bias-corrected 95% CI).

**Hyp.**	**Mediation Path**	**Indirect Effect**	**95% CI LL**	**95% CI UL**	**Significant?**
Panel A: Simple Mediation via Experience Perception (H9)					
H9	S1→O1→R1	0.105	0.062	0.158	Yes
H9	S2→O1→R1	0.086	0.041	0.139	Yes
H9	S3→O1→R1	0.142	0.092	0.204	Yes
Panel B: Simple Mediation via Emotional Attachment (H10)					
H10	S1→O2→R1	0.115	0.071	0.169	Yes
H10	S2→O2→R1	0.091	0.045	0.147	Yes
H10	S3→O2→R1	0.138	0.088	0.199	Yes
Panel C: Simple Mediation via Consumption Satisfaction (H9/H10 downstream)					
H9	S1→O3→R1	0.131	0.084	0.192	Yes
H9	S2→O3→R1	0.104	0.058	0.161	Yes
H9	S3→O3→R1	0.166	0.112	0.231	Yes
Panel D: Serial Mediation — O1→O2/O3→R (H11)					
H11	S3→O1→O3→R1	0.098	0.055	0.152	Yes
H11	S1→O1→O2→R1	0.072	0.039	0.118	Yes

Indirect effects are standardized. CI LL, confidence interval lower limit; CI UL, confidence interval upper limit. All reported indirect effects are significant (CI excludes zero). H9, mediation via Experience Perception (O1); H10, mediation via Emotional Attachment (O2); H11, serial mediation. Results for R2 (Traffic Circulation Behavior) follow the same pattern and are available from the authors upon request.

**Figure 3:**
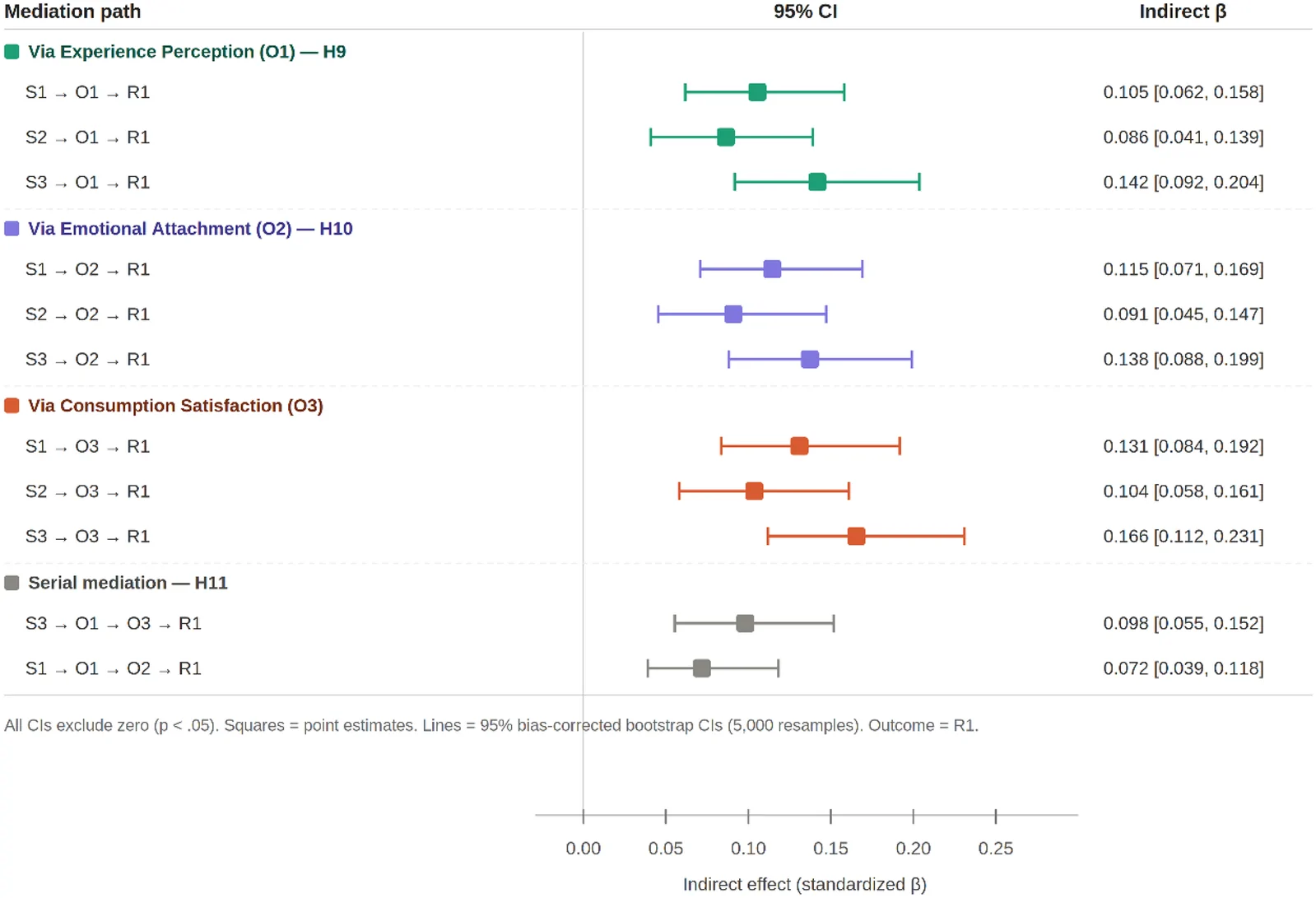
Forest plot of indirect (mediation) effects with 95% biaS,corrected bootstrap confidence intervals (*N* = 382,5,000 resamples). Squares indicate point estimates of standardized indirect effects; horizontal lines indicate 95% confidence intervals. All confidence intervals exclude zero, indicating statistically significant mediation. H9 = mediation via Experience Perception (O1); H10 = mediation via Emotional Attachment (O2); H11 = serial mediation. Outcome variable for all paths = Re-consumption Intention (R1).

#### 3.4.1 Simple mediation via experience perception (H9)

Experience Perception (O1) significantly mediated the effects of all three stimulus dimensions on Re-consumption Intention (R1). The indirect effects were 0.105 [95% CI (0.062, 0.158)] for Scene Immersion, 0.086 [95% CI (0.041, 0.139)] for Industry Integration, and 0.142 [95% CI (0.092, 0.204)] for Digital Services. All confidence intervals excluded zero, providing support for H9. Digital Services produced the largest indirect effect via Experience Perception, consistent with its status as the strongest predictor of O1 in the structural model.

##### 3.4.1.1 Simple mediation via emotional attachment (H10)

Emotional Attachment (O2) also significantly mediated the stimulus-to-response relationships. Indirect effects through O2 were 0.115 [95% CI (0.071, 0.169)] for Scene Immersion, 0.091 [95% CI (0.045, 0.147)] for Industry Integration, and 0.138 [95% CI (0.088, 0.199)] for Digital Services, all with confidence intervals excluding zero, supporting H10. Notably, the indirect effects via Emotional Attachment were consistently larger than those via Experience Perception for Scene Immersion and roughly comparable for Digital Services, underscoring the role of affective bonding as a particularly important mediating mechanism in the Su Chao consumer behavior chain.

##### 3.4.1.2 Mediation via consumption satisfaction

The indirect effects of stimulus dimensions on Re-consumption Intention via Consumption Satisfaction (O3) were the largest among the three simple mediation pathways. Indirect effects were 0.131 [95% CI (0.084, 0.192)] for Scene Immersion, 0.104 [95% CI (0.058, 0.161)] for Industry Integration, and 0.166 [95% CI (0.112, 0.231)] for Digital Services. The magnitude of these indirect effects reflects O3's position as the most proximal organism predictor of behavioral response in the structural model (*β* = 0.45 for R1), amplifying the downstream effects of all stimulus dimensions.

##### 3.4.1.3 Serial mediation (H11)

Two serial mediation chains were tested to evaluate H11. The path S3→O1→O3→R1 yielded a significant serial indirect effect of 0.098 [95% CI (0.055, 0.152)], confirming that the effect of Digital Services on Re-consumption Intention is sequentially transmitted through Experience Perception and Consumption Satisfaction. The path S1→O1→O2→R1 produced a significant serial indirect effect of 0.072 [95% CI (0.039, 0.118)], demonstrating that Scene Immersion's effect on Re-consumption Intention also operates through the sequential cognitive-to-affective chain. Both confidence intervals excluded zero, providing support for H11 and confirming the theoretical proposition that the organism tier operates as a sequential mediating system rather than a set of independent parallel channels.

Taken together, the mediation results reveal that the effects of the Su Chao consumption environment on consumer behavioral responses are substantially and consistently channeled through organism-level psychological processes. No stimulus dimension exerts a purely direct effect on behavioral response; all effects are fully or partially transmitted via the cognitive-affective-evaluative mediation chain, consistent with the theoretical architecture of the S-O-R framework.

To rigorously confirm the presence of “full mediation,” a direct-effects model was assessed. Crucially, when the organism variables (Experience Perception, Emotional Attachment, and Consumption Satisfaction) were simultaneously included in the model alongside the stimulus dimensions, the direct paths from all four stimulus variables (S1, S2, S3, S4) to the behavioral responses (R1, R2) became statistically non-significant (*p* > .05). This confirms that the behavioral impacts of the Su Chao consumption environment are fully mediated by the consumer's internal cognitive and affective states, adhering to the S-O-R framework's theoretical assumptions.

### 3.5 Robustness checks

Two supplementary analyses were conducted to assess the stability of the main findings. First, the structural model was re-estimated with four demographic covariates added as exogenous predictors of all organism and response variables: gender, age, monthly income, and event participation frequency. All 22 focal paths remained significant with effect sizes within ±0.03 of the baseline estimates. Among the control variables, participation frequency was a significant positive predictor of Re-consumption Intention (*β* = 0.14, *p* < .01) and Emotional Attachment (*β* = 0.11, *p* < .05), and age marginally predicted Consumption Satisfaction (*β* = 0.09, *p* < .05); gender and income were non-significant. Model fit remained acceptable ($\chi^{2}/df=2.71$, CFI = 0.94, TLI = 0.93, RMSEA = 0.053, SRMR = 0.046).

Second, the marker variable technique (45) was applied as a complement to the Harman single-factor test. Partial correlations controlling for the marker variable (respondents’ self-reported frequency of cooking at home) did not meaningfully differ from zero-order correlations, and the pattern of significant structural paths was unchanged. Combined with the Harman test result that the first unrotated factor accounted for only 28.4% of total variance, these checks provide reasonable assurance that common method variance does not threaten the validity of the present findings.

### 3.6 Moderation analysis (multi-group SEM)

To test the moderation hypotheses (H12 and H13), a Multi-Group Structural Equation Modeling (MG-SEM) procedure was employed. We sequentially tested configural invariance (unconstrained), metric invariance (factor loadings constrained), and scalar invariance (intercepts constrained). As shown in Table 8, the changes in fit indices between nested models (*Δ*CFI < 0.010) established sufficient measurement invariance to proceed with structural path comparisons (42).

**Table 8:** Multi-Group SEM invariance tests and moderation results.

Invariance Model	*χ* ^2^	df	CFI	RMSEA	Model Comparison	*Δ*χ^2^	*Δ*df	*Δ*CFI
H12: Participation Freq (Low vs. High)								
Configural Invariance	642.15	240	0.948	0.052	-	-	-	-
Metric Invariance	655.20	255	0.945	0.053	Metric vs. Configural	13.05	15	0.003
Scalar Invariance	670.82	270	0.941	0.054	Scalar vs. Metric	15.62	15	0.004
Structural Path Comparison (O2 → R1)					Low (β = 0.26) vs. High (β = 0.48)	6.84**	1	
H13: Age Cohort (Younger vs. Older)								
Configural Invariance	650.32	240	0.946	0.053	-	-	-	-
Metric Invariance	664.18	255	0.943	0.053	Metric vs. Configural	13.86	15	0.003
Scalar Invariance	679.55	270	0.939	0.055	Scalar vs. Metric	15.37	15	0.004
Structural Path Comparison (S3 → O1)					Younger (β = 0.45) vs. Older (β = 0.22)	8.12**	1	

**p < .01.

For H12, the path from Emotional Attachment (O2) to Re-consumption Intention (R1) was compared between low-frequency (≤1 time) and high-frequency (≥2 times) participants. The path coefficient was significantly stronger for high-frequency participants (*β* = 0.48, *p* < .001) compared to low-frequency participants (*β* = 0.26, *p* < .01), with a significant chi-square difference [*Δχ*^2^(1) = 6.84, *p* < .01]. This supports H12, indicating that repeated engagement solidifies the translation of affective bonds into future behavioral intent.

For H13, the path from Digital Services (S3) to Experience Perception (O1) was compared between younger (18–35) and older (≥36) cohorts. The positive effect of digital services was significantly stronger for younger consumers (*β* = 0.45, *p* < .001) than for older consumers (*β* = 0.22, *p* < .01) [*Δχ*^2^(1) = 8.12, *p* < .01], fully supporting H13.

## 4 Discussion

### 4.1 Main findings

Drawing on the S-O-R framework and data collected from 382 Su Chao consumers, this study examined 22 direct structural paths, nine mediation hypotheses, and two moderation effects. Overall, the findings are consistent with the proposed conceptual framework and provide a coherent account of the psychological processes linking the Su Chao consumption environment to consumer behavioral responses. Notably, all 22 hypotheses were supported. We attribute this pattern partly to the contextual characteristics of Su Chao's inaugural season. As a highly publicized grassroots sporting event featuring strong civic rivalry and extensive media exposure, the inaugural season generated relatively homogeneous consumer responses, which may have strengthened the observed structural relationships. These findings further suggest that the distinctive institutional and cultural characteristics of the Su Chao League shape consumer responses beyond those typically examined in conventional sports consumption settings.

The stimulus dimensions exerted differentiated effects across the organism variables. Digital Services emerged as the strongest predictor of both Experience Perception and Emotional Attachment, suggesting that for a predominantly young audience, interactive digital engagement and seamless online experiences have become central components of sports consumption rather than supplementary services. Scene Immersion exerted the strongest direct effect on Consumption Satisfaction, indicating that integrating local cultural symbols, civic identity, and immersive match-day experiences effectively enhances consumers’ hedonic evaluations. In contrast, Basic Facilities exhibited the weakest effects across all organism variables. This finding is consistent with Herzberg's two-factor theory, suggesting that basic physical facilities primarily function as hygiene factors that prevent dissatisfaction but are insufficient to generate strong emotional attachment or sustained behavioral engagement.

The organism tier operated consistently as a sequential cognitive-affective-evaluative system. Cognitive quality appraisals reliably preceded and shaped affective bonding, which sequentially finalized the evaluative judgment of satisfaction. Consumption Satisfaction functioned as the dominant proximal driver of behavioral response. However, the influence of Emotional Attachment was remarkably strong regarding Traffic Circulation Behavior. This suggests a distinct behavioral mechanism: while re-consumption is driven by overall satisfaction (a cost-benefit calculus), social diffusion and advocacy are acts of identity expression driven heavily by emotional bonding with the “one-city-one-team” ethos.

### 4.2 Theoretical implications

The findings provide several theoretical implications for extending the application of the S-O-R framework to grassroots sports consumption.

First, this study extends and contextualizes the stimulus dimension of the S-O-R framework within non-professional sporting event settings. Previous studies have predominantly conceptualized environmental stimuli in terms of generic servicescapes, such as stadium facilities, physical layout, and service quality, which adequately explain conventional commercial sports consumption but are less suited to grassroots sporting events characterized by strong civic identity and cultural embeddedness. The Su Chao phenomenon cannot be fully explained through generic experiential consumption perspectives alone. By conceptualizing Scene Immersion and Industry Integration as context-specific stimulus dimensions, this study incorporates civic identity, cultural embeddedness, and sport-tourism integration into the S-O-R framework, thereby extending its applicability to place-based sporting events.
Second, this study deepens the understanding of emotional attachment as a driver of social diffusion behavior in grassroots sports consumption. The results suggest that emotional attachment predicts Traffic Circulation Behavior beyond its influence on re-consumption intention, indicating that identity-expressive advocacy may represent a behaviorally distinct mechanism alongside satisfaction-driven word-of-mouth. This finding enriches current understanding of how civic identity is translated into active consumer participation and social dissemination within non-professional sporting events.
Third, this study broadens the response dimension of the S-O-R framework by conceptualizing Traffic Circulation Behavior as a co-equal behavioral outcome alongside re-consumption intention. Defined as consumers’ active dissemination of event-related content through social media sharing, word-of-mouth recommendation, and peer mobilization, Traffic Circulation Behavior links individual psychological responses with broader attention circulation and traffic economy dynamics. This broader conceptualization provides a behavioral bridge between sports consumer behavior research and the emerging literature on attention-driven local economic development.
Fourth, the serial mediation results provide additional empirical support for the sequential organism process within a non-professional sporting event context. While the cognitive-affective-evaluative sequence has been well established in psychology and consumer behavior research, the present findings demonstrate that this process also operates within grassroots sports consumption. Specifically, cognitive quality appraisals precede and shape emotional attachment, which subsequently contributes to consumption satisfaction. These findings provide context-specific evidence supporting the sequential processing mechanism proposed by the S-O-R framework and suggest that improving consumers’ initial cognitive evaluations may facilitate downstream emotional attachment and favorable behavioral outcomes.

### 4.3 Practical implications

The findings provide practical guidance for Su Chao organizers and other non-professional sports event managers seeking to foster sustainable consumer engagement. First, digital ecosystems should be treated as primary consumption environments rather than auxiliary service platforms. As Digital Services emerged as the strongest predictor of both Experience Perception and Emotional Attachment, investments should prioritize interactive live-streaming, seamless social sharing, personalized digital services, and online community engagement to strengthen both cognitive evaluations and emotional connections.

Second, organizers should leverage cultural embeddedness to differentiate the consumer experience. Because Scene Immersion exerted the strongest direct influence on Consumption Satisfaction, systematically integrating local cultural heritage, municipal identity, and the “one-city-one-team” rivalry into match-day experiences can effectively enhance emotional resonance and overall satisfaction. Finally, sport–tourism integration should be strategically developed to generate sustained local value. The consistent effects of Industry Integration across the psychological process suggest that carefully designed collaborations among sporting events, tourism, cultural attractions, hospitality, and local retail can transform short-term match-day traffic into more sustained consumer engagement and local economic spillovers.

### 4.4 Limitations and future research

This study presents several limitations that offer avenues for future research. First, the cross-sectional survey design precludes the establishment of definitive causal relationships among variables. While the structural equations strongly support the consistency of the S-O-R theoretical sequence, longitudinal tracking is required to firmly establish causality. Second, the sample was drawn through a combination of official social media platforms and on-site intercept surveys. Consequently, the sample is disproportionately young and highly educated, which, while reflective of Su Chao's active digital audience, cannot be considered strictly representative of the entire host-city population. Future studies should employ stratified random sampling to capture a broader demographic cross-section.

Finally, data collection occurred during Su Chao's inaugural 2025 season. The overwhelming support for the structural paths may partly reflect a “novelty effect” or “honeymoon phase,” where intense media coverage and the freshness of the civic rivalry generated highly positive, unified consumer consensus. This inaugural context may have amplified the perceived importance of Scene Immersion and Digital Services. As the league matures and the novelty subsides, the predictive weight of these stimuli may shift, potentially elevating the importance of core match quality and basic facilities. Longitudinal studies tracking consumer sentiment across multiple seasons are necessary to verify the durability of the traffic re-circulation mechanism in non-professional sports.

## 5 Conclusion

This study examined the behavioral mechanisms through which the innovative consumption environment of the Su Chao non-professional football league drives consumer re-consumption intention and traffic circulation behavior. By developing and testing a four-dimensional S-O-R model calibrated to the distinctive sport-tourism-heritage-digital fusion characteristics of Su Chao, the study provides the first systematic micro-level behavioral account of the consumer psychology underlying one of the most economically consequential grassroots sports phenomena to emerge in China in recent years.

The central empirical contribution is the demonstration that the Su Chao consumption environment activates a sequential cognitive-affective-evaluative chain—from experience perception through emotional attachment to consumption satisfaction—that fully mediates the effects of all four stimulus dimensions on behavioral outcomes. The traffic re-circulation dynamic at the heart of Su Chao's economic model is not a product of spectacle or novelty alone but of a structured psychological process in which high-quality experiential stimuli systematically generate the organism states that motivate both repeat consumption and peer-directed advocacy. Among the stimulus dimensions, digital services and scene immersion emerged as the two most potent drivers, with digital services producing the largest effect on experience perception (*β* = 0.36) and emotional attachment (*β* = 0.33), and scene immersion generating the strongest direct effect on consumption satisfaction (*β* = 0.29). Within the organism tier, the path from experience perception to emotional attachment was particularly strong (*β* = 0.42), and consumption satisfaction emerged as the dominant proximal predictor of both re-consumption intention (*β* = 0.45) and traffic circulation behavior (*β* = 0.34). Bootstrap mediation analyses further confirmed that the largest indirect effects on re-consumption intention were transmitted via consumption satisfaction, with digital services producing the strongest mediated pathway [indirect effect = 0.166, 95% CI (0.112, 0.231)].

The theoretical contributions are fourfold. First, the four-dimensional stimulus operationalization supersedes the unidimensional services cape conceptualizations of prior sports event S-O-R research. Second, the incorporation of traffic circulation behavior as a co-equal response variable connects sports consumer behavior research to the emerging literature on attention economy dynamics, establishing a behavioral foundation for traffic economy concepts that have been theorized at the industry level but not previously tested at the individual consumer level. Third, the three-stage sequential organism structure provides empirical support for a dual-process architecture in which cognitive evaluation precedes affective bonding, which in turn informs integrative satisfaction. Fourth, the moderation findings contribute to the literature on consumer heterogeneity in non-professional sports, underscoring the need for differentiated engagement strategies across demographic segments.

As non-professional sports leagues continue to emerge as significant drivers of local economic activity and social cohesion, understanding how innovative consumption environments generate sustainable consumer engagement becomes an increasingly pressing priority for sports economics research. The S-O-R framework developed and tested in this study offers a transferable analytical architecture for pursuing that agenda.

## Data Availability

The raw data supporting the conclusions of this article will be made available by the authors, without undue reservation.

## References

[1] Misener L Mason DS. Creating community networks: can sporting events offer meaningful sources of social capital? Managing Leisure. (2006) 11(1):39–56. 10.1080/13606710500445676

[2] Taks M Chalip L Green BC. Impacts and strategic outcomes from non-mega international sport events for local communities. Eur Sport Manag Q. (2015) 15(1):1–6. 10.1080/16184742.2014.995116

[3] Zhang D Zhang X. The intrinsic mechanism, operational logic, and practical path of the Jiangsu provincial urban football league in activating sports consumption potential. J Beijing Sport Univ. (2025) 48(7):32–9. in Chinese.

[4] Gu P Liu L Xu R. Research on the formation mechanism of communication benefits of the Jiangsu provincial urban football league. J Beijing Sport Univ. (2025) 48(7):3–12. in Chinese.

[5] Chen Y Chen L Zhou C Yin Z Gao Q. Innovative consumption scenarios promote the release of sports consumption potential: mechanisms, pathways, and strategies. J Shanghai Univ Sport. (2025) 49(10):13–26. in Chinese.

[6] Chai W Ye P. The mechanism and realization path of digital economy empowering sports industry chain resilience improvement. J Shandong Sport Univ. (2026) 42(1):44–54. 10.14104/j.cnki.1006-2076.2026.01.005. in Chinese.

[7] Wicker P Breuer C. Scarcity of resources in German non-profit sport clubs. Sport Manag Rev. (2011) 14(2):188–201. 10.1016/j.smr.2010.09.001

[8] Ding X Tian J Wang J. The value implications, realistic challenges, and promotion strategies for the development of urban football leagues in China. Sports Culture Guide. (2026) 1:64–71. in Chinese.

[9] Hightower R Brady MK Baker TL. Investigating the role of the physical environment in hedonic service consumption: an exploratory study of sporting events. J Bus Res. (2002) 55(9):697–707. 10.1016/S0148-2963(00)00211-3

[10] Wakefield KL Blodgett JG. The effect of the servicescape on customers’ behavioral intentions in leisure service settings. J Serv Mark. (1996) 10(6):45–61. 10.1108/08876049610148594

[11] Gibson HJ. Sport tourism: a critical analysis of research. Sport Manag Rev. (1998) 1(1):45–76. 10.1016/S1441-3523(98)70099-3

[12] Sui W Yu J Sun K. Ritualized construction of regional identity: how grassroots football events activate urban honor symbols — a case study of the Jiangsu provincial urban football league. Journal of Chengdu Sport University. (2025) 51(6):43–52. in Chinese.

[13] Mehrabian A Russell JA. An Approach to Environmental Psychology. Cambridge, MA: MIT Press (1974).

[14] Donovan RJ Rossiter JR. Store atmosphere: an environmental psychology approach. J Retail. (1982) 58(1):34–57.

[15] Eroglu SA Machleit KA Davis LM. Atmospheric qualities of online retailing: a conceptual model and implications. J Bus Res. (2001) 54(2):177–84. 10.1016/S0148-2963(99)00087-9

[16] Chan TKH Cheung CMK Lee ZWY. The state of online impulse-buying research: a literature analysis. Inf Manag. (2017) 54(2):204–17. 10.1016/j.im.2016.06.001

[17] Zhang H Lu Y Gupta S Zhao L. What motivates customers to participate in social commerce? The impact of technological environments and virtual customer experiences. Information&Management. (2014) 51(8):1017–30. 10.1016/j.im.2014.07.005

[18] He Y Xu F La L Ding J Zhao H. Research on the driving mechanism of cultural tourism consumption based on S-O-R theory. Tourism Science. (2023) 37(1):116–32. in Chinese.

[19] Pang D Liu X Li N. The influence mechanism of skiers’ willingness to spread online word-of-mouth: based on the S-O-R model. Era Economics and Trade. (2025) 22(9):76–80. in Chinese.

[20] Liu L Shi X. Influence mechanism of sport tourism consumption behavior under the background of COVID-19. Tourism Tribune. (2021) 36(8):52–70. in Chinese.

[21] Jia P Xu C Li X. Research on shopping behavioral intention in ethnic tourism scenic spots: based on the S-O-R theory. Social Scientist. (2022) 4:53–9. in Chinese.

[22] Yoshida M James JD. Customer satisfaction with game and service experiences: antecedents and consequences. Journal of Sport Management. (2010) 24(3):338–61. 10.1123/jsm.24.3.338

[23] Hur Y Ko YJ Valacich J. A structural model of the relationships between sport website quality, e-satisfaction, and e-loyalty. Journal of Sport Management. (2011) 25(5):458–74. 10.1123/jsm.25.5.458

[24] Zeithaml VA Parasuraman A Malhotra A. Service quality delivery through web sites: a critical review of extant knowledge. J Acad Mark Sci. (2002) 30(4):362–75. 10.1177/009207002236911

[25] Brady MK Cronin JJ. Some new thoughts on conceptualizing perceived service quality: a hierarchical approach. J Mark. (2001) 65(3):34–49. 10.1509/jmkg.65.3.34.18334

[26] Lazarus RS. Emotion and Adaptation. New York, NY: Oxford University Press (1991).

[27] Parasuraman A Zeithaml VA Berry LL. SERVQUAL: a multiple-item scale for measuring consumer perceptions of service quality. J Retail. (1988) 64(1):12–40.

[28] Pine BJ Gilmore JH. The Experience Economy: Work is Theatre and Every Business a Stage. Boston, MA: Harvard Business School Press (1999).

[29] Altman I. Low S. M. (Eds.). Place Attachment. New York, NY: Plenum Press (1992). xix+314 pp.

[30] Park CW MacInnis DJ Priester J Eisingerich AB Iacobucci D. Brand attachment and brand attitude strength: conceptual and empirical differentiation of two critical brand equity drivers. J Mark. (2010) 74(6):1–17. 10.1509/jmkg.74.6.1

[31] Funk DC James JD. The psychological continuum model: a conceptual framework for understanding an individual’s psychological connection to sport. J Sport Manag. (2001) 15(2):119–50. 10.1016/S1441-3523(01)70072-1

[32] Oliver RL. Cognitive, affective, and attribute bases of the satisfaction response. J Consum Res. (1993) 20(3):418–30. 10.1086/209358

[33] Theodorakis ND Alexandris K Tsigilis N Karvounis S. Predicting spectators’ behavioural intentions in professional football: the role of satisfaction and service quality. Sport Manag Rev. (2013) 16(1):85–96. 10.1016/j.smr.2012.05.004

[34] Cheng L Yuan Y. Research on the impact of multi-dimensional tourist experience on behavioral intentions in historical and cultural blocks. J Sichuan Admin Coll. (2026) 2026(2):40–52. Advance online publication. (in Chinese).

[35] Cheng J Huang Y. Research on the impact of new retail experience on consumer post-purchase behavior: the mediating role of psychological distance. Comm Econ Res. (2024) 23:60–3. in Chinese.

[36] Li K Guo K. The influence of social distance on consumers’ willingness to spread word-of-mouth in social media marketing contexts. Comm Econ Res. (2025) 13:81–4. in Chinese.

[37] Hair JF Black WC Babin BJ Anderson RE. Multivariate Data Analysis (8th ed.). Andover, Hampshire: Cengage (2019).

[38] Anderson JC Gerbing DW. Structural equation modeling in practice: a review and recommended two-step approach. Psychol Bull. (1988) 103(3):411–23. 10.1037/0033-2909.103.3.411

[39] Hu L Bentler PM. Cutoff criteria for fit indexes in covariance structure analysis: conventional criteria versus new alternatives. Struct Equ Modeling. (1999) 6(1):1–55. 10.1080/10705519909540118

[40] Fornell C Larcker DF. Evaluating structural equation models with unobservable variables and measurement error. J Mark Res. (1981) 18(1):39–50. 10.1177/002224378101800104

[41] Preacher KJ Hayes AF. Asymptotic and resampling strategies for assessing and comparing indirect effects in multiple mediator models. Behav Res Methods. (2008) 40(3):879–91. 10.3758/BRM.40.3.879 18697684

[42] Cheung GW Rensvold RB. Evaluating goodness-of-fit indexes for testing measurement invariance. Struct Equ Modeling. (2002) 9(2):233–55. 10.1207/S15328007SEM0902_5

[43] Podsakoff PM MacKenzie SB Lee J-Y Podsakoff NP. Common method biases in behavioral research: a critical review of the literature and recommended remedies. J Appl Psychol. (2003) 88(5):879–903. 10.1037/0021-9010.88.5.879 14516251

[44] Nunnally JC. Psychometric Theory (2nd ed.). New York, NY: McGraw-Hill (1978).

[45] Lindell MK Whitney DJ. Accounting for common method variance in cross-sectional research designs. J Appl Psychol. (2001) 86(1):114–21. 10.1037/0021-9010.86.1.114 11302223

