# Supplementary material for: Consumption environment, fan engagement, and social diffusion in non-professional football: an S-O-R study of China's Su Chao league
Source: Front Sports Act Living. 2026 Oct 8;8:1868151. doi: 10.3389/fspor.2026.1868151 (PMC13687059; doi:10.3389/fspor.2026.1868151)
Supplement: Supplementary file 1 [file Supplementaryfile1.docx]

**Questionnaire on the Influence Mechanism of the Suzhou Super League (SSL) Innovative Consumption Space on Consumers’ Repurchase Intentions**

**[Informed Consent]**

Dear Participant,

We are a research team from Nanjing University specializing in sports industry and consumer behavior. This survey is an academic research project aimed at exploring the influence mechanism of innovative consumption spaces in regional non-professional football events on consumers’ repurchase behavior. All research procedures strictly comply with the ethical principles of the Declaration of Helsinki.

**Rights and Information:**

1. This questionnaire is completely anonymous. All data will be used exclusively for academic research. No personally identifiable information will be collected, nor will any data be disclosed to any third party or used for commercial purposes.

2. The questionnaire takes approximately 6–8 minutes to complete. You may unconditionally withdraw at any time and have the right to request the withdrawal of all your data within one month after submission by contacting the reserved email address.

3. All survey data will be encrypted and stored on the research team’s dedicated academic server. The data will be permanently archived for five years after publication of the research findings, after which it will be securely destroyed.

4. This questionnaire contains no risk items. There are no right or wrong answers; please respond based on your genuine feelings and actual experiences.

By submitting this questionnaire, you acknowledge that you have been fully informed of the above and voluntarily consent to participate in this academic research without any coercion. Thank you sincerely for your support and cooperation!

**[Instructions]**

1. This questionnaire consists of four modules. The item order has been randomized to avoid order effects. Please answer all items in the order presented and do not skip any items.

2. Unless otherwise noted, all core items use a 5-point Likert scale: 1 = Strongly Disagree, 2 = Disagree, 3 = Neutral, 4 = Agree, 5 = Strongly Agree.

3. Please complete the questionnaire independently without discussing with others to ensure the authenticity and validity of the data.

4. This questionnaire aims to capture your genuine experience. Please respond objectively based on your actual feelings, including neutral or negative evaluations.

**[Part 1: Demographic Characteristics and Control Variables]**

Please select the option that best describes your situation.

1. Gender: □ Male □ Female □ Other

2. Age: □ 18 or below □ 19–25 □ 26–35 □ 36–45 □ 46 or above

3. Highest level of education: □ High school or below □ Associate degree □ Bachelor’s degree □ Master’s degree or above

4. Average monthly disposable income (CNY): □ 3,000 or below □ 3,001–5,000 □ 5,001–8,000 □ 8,001–12,000 □ 12,001 or above

5. Frequency of participation in SSL-related activities: □ Never participated (online awareness only) □ Once □ 2–3 times □ 4–6 times □ 7 times or more

6. Average per-person spending per SSL participation: □ 100 CNY or below □ 101–300 CNY □ 301–500 CNY □ 501–1,000 CNY □ 1,001 CNY or above

7. Primary mode(s) of SSL participation (multiple selections allowed): □ On-site spectating □ Online live streaming □ Offline derivative consumption (merchandise, cultural tourism integration, etc.) □ Online community interaction □ Other ____

8. Frequency of football-related sports/activities in the past year: □ Never □ 1–2 times □ 3–5 times □ 6–10 times □ 11 times or more

9. Familiarity with domestic non-professional football leagues: □ Not familiar at all □ Not very familiar □ Somewhat familiar □ Fairly familiar □ Very familiar

**[Part 2: Stimulus (S) — External Environment]**

**▶ Scene Immersion (S1)**

1. The SSL events create an attractive immersive spectating atmosphere. □ 1 □ 2 □ 3 □ 4 □ 5

2. The SSL’s integration of local cultural elements enhances the uniqueness of the overall experience. □ 1 □ 2 □ 3 □ 4 □ 5

3. The SSL provides fans with diverse interactive participation scenarios. □ 1 □ 2 □ 3 □ 4 □ 5

**▶ Industry Convergence (S2)**

4. The SSL offers a comprehensive consumption experience combining “spectating + cultural tourism.” □ 1 □ 2 □ 3 □ 4 □ 5

5. The SSL effectively integrates sports with local culture (e.g., intangible cultural heritage). □ 1 □ 2 □ 3 □ 4 □ 5

6. The SSL’s derivative products are attractive. □ 1 □ 2 □ 3 □ 4 □ 5

**▶ Digital Services (S3)**

7. The SSL’s online spectating and ticketing processes are convenient and efficient. □ 1 □ 2 □ 3 □ 4 □ 5

8. The SSL’s online interaction and functional design are well-developed. □ 1 □ 2 □ 3 □ 4 □ 5

9. The SSL’s digital services enhance the overall experience. □ 1 □ 2 □ 3 □ 4 □ 5

**▶ Basic Support (S4)**

10. The SSL provides a safe and reliable spectating and consumption environment. □ 1 □ 2 □ 3 □ 4 □ 5

11. The SSL’s service staff demonstrate a high level of professionalism. □ 1 □ 2 □ 3 □ 4 □ 5

12. The SSL’s pricing for tickets and products is reasonable. □ 1 □ 2 □ 3 □ 4 □ 5

13. The SSL’s basic services and supporting facilities have NOT adequately met my basic needs. (Reverse-scored) □ 1 □ 2 □ 3 □ 4 □ 5

**[Part 3: Organism (O) — Psychological Mechanism]**

**▶ Experience Perception (O1)**

14. The SSL’s overall experience is rich and attractive. □ 1 □ 2 □ 3 □ 4 □ 5

15. The SSL’s various services are well-connected and convenient to use. □ 1 □ 2 □ 3 □ 4 □ 5

16. The SSL’s consumption experience is distinctly unique. □ 1 □ 2 □ 3 □ 4 □ 5

17. The SSL’s overall experience offers good value for money. □ 1 □ 2 □ 3 □ 4 □ 5

18. The SSL’s overall consumption experience falls significantly short of my core needs. (Reverse-scored) □ 1 □ 2 □ 3 □ 4 □ 5

**▶ Emotional Attachment (O2)**

19. Participating in SSL events has strengthened my sense of identification with the city. □ 1 □ 2 □ 3 □ 4 □ 5

20. I have developed a positive emotional identification with the SSL events. □ 1 □ 2 □ 3 □ 4 □ 5

21. Participating in the SSL makes me feel happy and engaged. □ 1 □ 2 □ 3 □ 4 □ 5

22. I hold a positive emotional attitude toward the SSL brand. □ 1 □ 2 □ 3 □ 4 □ 5

**▶ Consumer Satisfaction (O3)**

23. The SSL’s overall experience meets my expectations. □ 1 □ 2 □ 3 □ 4 □ 5

24. I am satisfied with the quality of services provided by the SSL. □ 1 □ 2 □ 3 □ 4 □ 5

25. Overall, I am satisfied with my SSL experience. □ 1 □ 2 □ 3 □ 4 □ 5

**[Part 4: Response (R) — Behavioral Outcomes]**

**▶ Repurchase Intention (R1)**

26. I am willing to participate in SSL-related activities again. □ 1 □ 2 □ 3 □ 4 □ 5

27. I am willing to continue following SSL event information. □ 1 □ 2 □ 3 □ 4 □ 5

28. I am willing to purchase SSL-related products or services again. □ 1 □ 2 □ 3 □ 4 □ 5

**▶ Traffic Circulation Behavior (R2)**

29. I am willing to recommend the SSL experience to others. □ 1 □ 2 □ 3 □ 4 □ 5

30. I am willing to share my experience on social media. □ 1 □ 2 □ 3 □ 4 □ 5

31. I am willing to encourage others to participate in SSL activities. □ 1 □ 2 □ 3 □ 4 □ 5

**Scale Specification Notes**

**(I) Core Framework and Construct Definitions**

This scale is constructed based on the classical S-O-R (Stimulus–Organism–Response) theory, adopting a reflective measurement model. It comprises 3 first-order latent variables, 9 second-order latent variables, and a total of 32 items (including 2 reverse-scored items).

| **First-Order Latent Variable** | **Second-Order Latent Variable** | **Theoretical Definition of Construct** | **No. of Items** |
| --- | --- | --- | --- |
| Stimulus (S) — Exogenous Latent Variable | Scene Immersion Stimulus | Objective scene characteristics of the SSL event consumption space that trigger immersive experiences for consumers, including on-site spectating atmosphere, online virtual experiences, cultural element integration, and community scene construction. | 4 |
|  | Industry Convergence Stimulus | Objective characteristics of the diversified and differentiated experiential offerings provided to consumers through the SSL’s “Sports + Cultural Tourism + Intangible Cultural Heritage + Digitalization” convergence model. | 4 |
|  | Digital Service Stimulus | Objective characteristics of digital services supporting online consumption and experience, including operational convenience, functional completeness, response efficiency, and security assurance. | 4 |
|  | Basic Support Stimulus | Objective characteristics of hardware and services supporting consumers’ basic experience, including safety assurance, service quality, pricing reasonableness, and facility completeness. | 5 (incl. 1 reverse) |
| Organism (O) — Endogenous Mediating Latent Variable | Experience Perception | Consumers’ rational cognitive evaluations of the overall characteristics of the SSL innovative consumption space, serving as the direct cognitive outcome of external stimuli. | 5 (incl. 1 reverse) |
|  | Emotional Attachment | Emotional attachment, identification, and positive emotional responses formed by consumers toward the event brand and associated culture during the SSL consumption experience; an emotional extension of cognitive evaluation. | 4 |
|  | Consumer Satisfaction | Consumers’ comprehensive emotional evaluation of the gap between the overall SSL consumption experience and prior expectations; an integrated outcome of cognition and emotion that directly drives behavioral responses. | 4 |
| Response (R) — Endogenous Outcome Latent Variable | Repurchase Intention | Consumers’ subjective behavioral tendency to participate in SSL spectating and consumption again in the future, driven by psychological states; the core dependent variable of this study. | 3 |
|  | Traffic Circulation Behavior | Consumers’ proactive positive dissemination behaviors including word-of-mouth recommendation, social sharing, and encouraging others to participate; a core behavioral outcome driving event traffic appreciation and closed-loop circulation. | 3 |

**(II) Scoring and Data Processing Specifications**

1. Positively worded items: Use the raw score (1–5) as provided by respondents. Higher scores indicate stronger levels of the corresponding construct.

2. Reverse-scored items: Apply the standardized transformation formula: Transformed Score = 6 – Raw Score. After transformation, the scoring logic is fully consistent with positively worded items in the same dimension, ensuring unidirectionality of construct measurement.

3. Construct aggregation scoring: The aggregate score of each latent variable = the mean of all items in that dimension (including transformed reverse-scored items), used for subsequent descriptive statistics, regression analysis, and structural equation modeling (SEM) testing.

4. Reliability and validity testing criteria:

Reliability: Cronbach’s α ≥ 0.7 and composite reliability (CR) ≥ 0.7 for each latent variable, demonstrating good internal consistency.

Convergent validity: Average variance extracted (AVE) ≥ 0.5 for each latent variable, confirming adequate explanatory power of items for the latent variable.

Discriminant validity: Using the Fornell–Larcker criterion (the square root of each latent variable’s AVE exceeds its correlations with all other latent variables) or HTMT ratio < 0.85, confirming good discriminability among latent variables without serious construct overlap.

**(III) Common Method Variance (CMV) Control Measures**

1. Procedural controls: (a) Fully anonymous administration to reduce social desirability bias; (b) Randomized item order to avoid order effects; (c) Predictor variables (S) and outcome variables (R) are fully separated in the questionnaire by mediating variables (O), disrupting respondents’ consistency mindset; (d) All items use concise, unambiguous, neutral wording to avoid leading language.

2. Statistical controls: Both pilot and main study data will be subjected to Harman’s single-factor test. If the variance explained by the first unrotated factor is < 40%, this confirms the absence of serious common method variance. If bias exceeds the threshold, the Unmeasured Latent Method Construct (ULMC) approach will be applied for correction, fully complying with top-tier journal standards.

**III. In-Depth Interview Guide**

**[Interview Design Notes]**

This interview adopts an explanatory sequential mixed-methods design as a supplement and extension to the quantitative research. Its core purpose is to explain the quantitative results, particularly significant/non-significant path relationships and heterogeneous outcomes, and to uncover deep mechanisms and contextual boundaries that quantitative data cannot reveal. All content strictly follows the COREQ (Consolidated Criteria for Reporting Qualitative Research) standards.

**[Core Interview Information]**

Interview duration: 25–35 minutes per person

Sampling strategy: Maximum variation sampling, covering consumers with varying participation frequencies, spending levels, and participation modes, as well as event operators and industry experts, to ensure sample heterogeneity.

Ethical standards: Written informed consent signed before the interview, clearly informing participants of the interview purpose, audio recording usage, anonymization procedures, and data withdrawal rights. All interview materials are used exclusively for academic research.

Analytical method: Six-step thematic analysis using NVivo 12 software for coding analysis, ensuring rigor and reproducibility.

**(I) Consumer In-Depth Interview Guide**

Interviewees: SSL event consumers (differentiated by first-time vs. repeat participants, high vs. low spenders, online vs. offline participants)

**Warm-up Questions (Building rapport and establishing interview boundaries)**

1. Hello, thank you very much for accepting this interview. First, could you briefly describe how you learned about the SSL events? How many times have you participated in SSL-related spectating/consumption activities?

2. When participating in sports events or leisure consumption activities, what are the core factors you value most?

**Core Question Set 1: Consumption Scene Experience (Corresponding to the S-Stimulus dimension in quantitative research)**

1. Among all your SSL experiences, which scene or segment left the deepest impression? What was that scene specifically like? How did it make you feel?

2. Have you personally experienced the SSL’s “Sports + Cultural Tourism + Intangible Cultural Heritage” convergence model? How does this model differ from other events in your opinion? What is its appeal to you?

3. Regarding the SSL’s digital online services (online spectating, interaction, ticketing, etc.), what aspects do you think work well, and where are there notable shortcomings? Can you give a specific example?

4. What do you consider the biggest barrier to participating in SSL-related activities (e.g., time, cost, transportation, information access)? What factors limit your participation?

**Core Question Set 2: Psychological Perception and Emotional Attachment (Corresponding to the O-Organism dimension)**

1. After participating in SSL-related activities, what is the most central impression you formed of the SSL as an event brand?

2. Did the incorporation of local urban culture and intangible cultural heritage elements make you feel differently about the event or the local city? Why did you feel that way?

3. Overall, are you satisfied with your SSL consumption experience? Compared to your initial expectations, what aspects exceeded expectations and what fell short?

4. Was there any experience that caused you to develop strong positive or negative feelings toward the SSL? What was that experience specifically?

**Core Question Set 3: Repurchase and Dissemination Behavior (Corresponding to the R-Response dimension)**

1. Are you willing to participate in SSL spectating/consumption activities again in the future? What are the three most critical factors influencing your decision?

2. Have you recommended the SSL to people around you? If so, what motivated you to recommend it? If not, what are the reasons you chose not to?

3. In your opinion, what is the core issue the SSL needs to address to encourage more people to participate repeatedly and recommend it proactively?

**Closing Questions**

1. Do you have any other suggestions for the SSL’s future consumption scene development and event operations?

2. Is there anything else you would like to add regarding what we discussed today?

**(II) SSL Operations Team In-Depth Interview Guide**

Interviewees: Core SSL operations managers, commercial partnership directors, consumption scene designers, traffic operations managers

**Warm-up Question**

1. Hello, thank you very much for accepting this interview. First, could you briefly introduce your core responsibilities in SSL event operations and the key development milestones of the SSL project from inception to present?

**Core Question Set 1: Scene Design Logic and Positioning**

1. What is the core positioning and design philosophy behind the SSL’s creation of an innovative consumption space? Compared to traditional non-professional football leagues, what key differentiation breakthrough are you aiming to achieve?

2. What consumer needs informed the current SSL consumption scene layout (on-site spectating, cultural tourism integration, intangible cultural heritage interaction, digital services)? What consumer research and needs validation were conducted during the design process?

3. What was the biggest challenge in implementing the consumption scenes, and how was it resolved?

**Core Question Set 2: Operational Status and Consumer Feedback**

1. Based on operational data, what is the current core consumer profile for the SSL? Do consumer repurchase rates and word-of-mouth effects meet expectations? Which metrics exceeded expectations, and which fell short?

2. During operations, what are the main areas of positive consumer feedback? Where are negative feedback and complaints concentrated? What optimizations have been made in response?

3. What differentiated operational strategies have been designed for different consumer types (core fans, casual leisure consumers, cultural tourism consumers)? How effective have they been?

**Core Question Set 3: Traffic Circulation and Sustainable Operations**

1. What core strategies does the SSL currently employ to encourage consumer repurchase and sustain traffic circulation? Which strategies have been most effective?

2. How do you view the “traffic attraction → retention → value appreciation” closed-loop model for non-professional football events? What core layouts and plans has the SSL developed for building this closed loop?

3. Over the next three years, what are the SSL’s core development plans for consumption space optimization, consumer experience enhancement, and sustainable operations?

**Closing Questions**

1. What do you consider the core challenges and opportunities for domestic non-professional sports events in building a sustainable consumption ecosystem?

2. Is there anything else you would like to add regarding what we discussed today?

**(III) Industry Expert In-Depth Interview Guide**

Interviewees: Sports management scholars, sports industry researchers, leading domestic non-professional event operations experts

**Warm-up Question**

1. Hello, thank you very much for accepting this interview. First, could you briefly introduce your core research or practice areas in the fields of sports industry, sports consumption research, or non-professional event operations?

**Core Question Set 1: Industry Development Trends**

1. In your view, what core trends are emerging in the consumption upgrade of domestic non-professional sports events? Compared to five years ago, what fundamental changes have occurred in consumer demands?

2. What are the common pain points and bottlenecks that domestic non-professional sports events face in creating consumption spaces and improving consumer repurchase rates?

3. How do you assess the value of the “Sports + Cultural Tourism + Intangible Cultural Heritage” convergence model in non-professional sports events? What are the critical success factors for this model to be implemented effectively?

**Core Question Set 2: Theoretical and Practical Alignment**

1. How well do you think the S-O-R theory fits in analyzing sports event consumption behavior, particularly the influence mechanism of innovative consumption spaces in non-professional events? What should be the core analytical dimensions?

2. From an academic research perspective, what innovative value and research significance do you see in the innovative consumption space practices of regional non-professional leagues like the SSL for the development of non-professional events?

3. What core research gaps currently exist in the academic literature on non-professional sports event consumption behavior?

**Core Question Set 3: Sustainable Development Pathways**

1. What do you consider the core leverage points for domestic non-professional sports events to achieve a virtuous business closed loop of “traffic aggregation → retention conversion → repurchase value appreciation”?

2. For regional non-professional leagues like the SSL seeking to further amplify the value of consumption spaces and achieve long-term sustainable development, what are your core recommendations?

**Closing Question**

1. Is there anything else you would like to add regarding the topics related to non-professional event consumption that we discussed today?
